# Efficacy of Lactoferrin Oral Administration in the Treatment of Anemia and Anemia of Inflammation in Pregnant and Non-pregnant Women: An Interventional Study

**DOI:** 10.3389/fimmu.2018.02123

**Published:** 2018-09-21

**Authors:** Maria Stefania Lepanto, Luigi Rosa, Antimo Cutone, Maria Pia Conte, Rosalba Paesano, Piera Valenti

**Affiliations:** ^1^Department of Public Health and Infectious Diseases, University of Rome La Sapienza, Rome, Italy; ^2^Department of Biosciences and Territory, University of Molise, Pesche, Italy; ^3^Department of Gynecological-Obstetric and Urological Sciences, University of Rome La Sapienza, Rome, Italy

**Keywords:** lactoferrin, iron, anemia, anemia of inflammation, β-thalassemia, hereditary thrombophilia

## Abstract

The discovery of the ferroportin-hepcidin complex has led to a critical review on the treatment of anemia and anemia of inflammation (AI). Ferroportin, the only known mammalian iron exporter from cells to blood, is negatively regulated by hepcidin, a hormone peptide able to bind to ferroportin, leading to its degradation. Therefore, new efficient therapeutic interventions acting on hepcidin and ferroportin are imperative to manage anemia and AI. Bovine milk derivative lactoferrin (bLf), a glycoprotein able to chelate two ferric ions per molecule, is emerging as a natural anti-inflammatory substance able to modulate hepcidin and ferroportin synthesis through the down-regulation of interleukin-6 (IL-6). Here, an interventional study (ClinicalTrials.gov Identifier: NCT01221844) was conducted by orally administering 100 mg of 20–30% iron-saturated bLf (corresponding to 70–84 μg of elemental iron) twice a day. This treatment was compared with the Italian standard therapy, consisting in the oral administration of 329.7 mg of ferrous sulfate once a day (corresponding to 105 mg of elemental iron). Treatments were carried out on 29 anemic women with minor β-thalassemia (20 pregnant and 9 non-pregnant), 149 women with hereditary thrombophilia (HT) (70 pregnant and 79 non-pregnant) affected by AI and 20 anemic pregnant women suffering from various pathologies. In anemic pregnant and non-pregnant women with minor β-thalassemia, presenting undetectable hepcidin levels, differently from ferrous sulfate management, bLf decreased IL-6 (from 25 ± 8 to 6 ± 3 pg/ml) and increased total serum iron (TSI) (from 54 ± 17 to 80 ± 9 μg/dl). BLf was also more efficient than ferrous sulfate in AI treatment in HT pregnant and non-pregnant women by decreasing both serum IL-6 (from 89 ± 8 to 58 ± 6 pg/ml) and hepcidin (from 115 ± 23 to 65 ± 10 ng/ml), thus increasing hematological parameters, such as the number of red blood cells (RBCs), the concentration of hemoglobin, TSI and serum ferritin. BLf was also efficient in treating anemia in other pathological pregnancies. Taken together all the results, bLf, showing a greater benefit and efficacy than the standard ferrous sulfate management, can be considered as a promising compound in treating anemia and AI through its ability to down-regulate IL-6, thus restoring ferroportin-mediated iron export from cells to blood in a hepcidin-dependent or independent way.

## Introduction

Iron is an essential nutrient for living cells and it is pivotal during pregnancy, in particular for the developing fetus through maternal iron transfer as well as in the neonate through breast-feeding and in the childhood through diet. Iron deficiency (ID) is considered the most important nutritional disorder in the world, being more prevalent in pregnant women where represents a high risk factor for maternal and infant health associated with preterm delivery, fetal growth retardation, low birth weight, and inferior neonatal health (1). ID is also frequent in children where is related to decreased brain functions (2, 3). Moreover, women of reproductive age can be affected by ID due to iron loss during the menses. An early diagnosis and a prompt management of ID and ID anemia (IDA) are highly recommended. ID is characterized by the low levels of total serum iron (TSI) and serum ferritin (sFtn). Conversely, IDA is characterized by low concentrations of hemoglobin (Hb) and low number of red blood cells (RBCs) in addition to TSI and sFtn low levels.

The human body contains 3–4 g of iron localized in hemoglobin of erythrocytes (about 2.0–2.5 g), in ferritin (Ftn) within hepatocytes and macrophages (about 0.5–1 g), in myoglobin, and in iron-containing enzymes (about 0.5 g). A minor iron source, deriving from an equilibrated diet, provides about 15 mg of iron per day of which only about 10%, corresponding to 1–2 mg, is absorbed due to its exceptionally poor bio-availability. A major iron source involves the recycling of iron from the lysis of senescent erythrocytes by macrophages (about 20 mg/day) (4, 5).

The correct balance of iron between tissues/secretions and blood, defined iron homeostasis, is regulated by two main factors, ferroportin (Fpn) and hepcidin (6, 7). Fpn is the only known mammalian iron exporter from tissues to blood (6) down-regulated by the pro-inflammatory interleukin (IL)-6 (8, 9). Hepcidin, a 25 amino acids cationic peptide hormone, identified in plasma (6) and urine (10, 11), is modulated by iron stores and it is up-regulated by pro-inflammatory cytokines, such as IL-6, IL-1α, and IL-1β (12–16).

Overall, iron homeostasis is highly regulated through three different pathways: iron absorption in apical side of enterocytes, iron storage into ferritin and iron export by Fpn (17, 18). Fpn, besides IL-6 and the amount of intracellular iron/heme, is regulated also by hepcidin which is able to bind to Fpn, leading to its internalization and degradation (19). Consequently, high hepcidin concentrations inhibit iron export from cells to blood while low levels allow iron export through Fpn (5, 20).

Interestingly, in the recent study by Willemetz et al. (21), it has been demonstrated, in a model of hepcidin knockout (Hamp^−/−^) murine macrophages infected by *Salmonella enterica*, that Fpn down-regulation can occur also in a hepcidin-independent manner (21).

The iron homeostasis disorders consist in the up-expression of hepcidin and the subsequent down-regulation of Fpn leading to anemia of inflammation (AI), a pathological condition characterized by low hematological parameters, normal-to-elevated sFtn, high levels of IL-6 and of other pro-inflammatory cytokines (22). These disorders are traditionally treated with iron supplementations. However, in papers by Paesano et al. (4) and Rosa et al. (23) the iron homeostasis disorders have not been defined as iron deficiency but as iron delocalization, characterized by iron overload in tissues/secretions and iron deficiency in blood. In a pathological condition occurring in patients affected by β-thalassemia, one of the types of thalassemia caused by a mutation in the β-globin gene (*HBB*) on chromosome 11, hepcidin production is suppressed (5, 20). The phenotypes of β-thalassemias include β-thalassemia major, intermedia and minor, according to the severity of the genetic mutation. Individuals with β-thalassemia major, characterized by marked ineffective erythropoiesis and severe hemolysis, as well as with β-thalassemia intermedia, characterized by widely variable symptoms and severity ranging between the two extremes of the major and minor forms, require frequent to occasional RBCs transfusions to sustain life (24–26). Among β-thalassemic (27, 28), the subjects affected by minor β-thalassemia, clinically asymptomatic and characterized by morphological abnormalities of erythrocytes as well as by a slight lowering of the hemoglobin levels, show the disorders of iron homeostasis sometimes leading to a mild anemia (24). During pregnancy, despite the progress of therapies, maternal and fetal complications, such as risk of abortion, thromboembolism, pre-term delivery and intrauterine growth restriction, appear to be increased in patients with β-thalassemia major, occurring in almost 40% of pregnancies, as well as in patients with β-thalassemia intermedia, occurring in 20–30% of pregnancies, compared to healthy ones (25, 26). Regarding β-thalassemia minor, the incidence of premature and low birth weight infants was found to be comparable to the general population. Of note, there is no specific therapy for β-thalassemia minor subjects during pregnancy, but if the anemia becomes more severe transfusions are sometimes necessary (25, 26).

Concerning the other pathology treated in this study, the hereditary thrombophilia (HT), there are no data in literature on the correlation between hepcidin levels and the establishing of AI in HT subjects. However, higher levels of serum IL-6 compared to healthy subjects have been described in HT pregnant women (29). HT is a genetic predisposition to the formation of venous thrombus due to coagulation abnormalities (30, 31). During pregnancy, this hypercoagulable state is exacerbated due to the increase of factors VII, VIII, X and von Willebrand factor activities and to marked increases in fibrinogen (32). Thrombin generation markers such as prothrombin F1 and F2 also increase (33). Moreover, activation of the coagulation system is characterized by the co-participation of inflammatory response components as IL-6, IL-8, and TNF-α (34). All these changes can predispose to several maternal and fetal complications during the pregnancy, including pre-eclampsia, late and recurrent early miscarriage, placental abruption, fetal growth restriction (FGR), and intrauterine death and stillbirth (33). For all these reasons, in order to avoid maternal and fetal sequelae, all HT pregnant women must undergo a proper therapeutic management.

An important component of iron and inflammatory homeostasis machinery is an iron binding glycoprotein, named lactoferrin (Lf), present in human secretions, like milk, saliva, vaginal fluid, amniotic fluid, upper airway fluid, seminal plasma, the cervical mucus, and earwax (23). Similar to proteins of the secretions, Lf is a multifunctional glycoprotein able to protect mucosa from the injury of microbial attachment and colonization as well as to exert anti-inflammatory activity (18, 35). The bovine milk derivative lactoferrin (bLf), generally recognized as safe (GRAS) by Food and Drug Administration (FDA-USA), is emerging as an important natural glycoprotein able to treat IDA and AI (4, 29, 36–38). The efficacy of bLf treatment is related to its ability to decrease IL-6 concentration which, in turn, modulates hepcidin and Fpn synthesis. Recent *in vitro* studies showed how high levels of IL-6 down-regulate the expression of Fpn in inflamed-macrophages and how bLf is able to efficiently revert this effect, down-regulating IL-6 and up-regulating Fpn (8, 9). These results demonstrate that bLf, able to decrease IL-6 levels, exerts a role in iron and inflammatory homeostasis and, especially, in treating AI.

Of note, previous *in vivo* studies have reported non-pregnant women affected by minor β-thalassemia to possess higher levels of IL-6 compared with non-pregnant healthy women (38). Conversely, pregnant women suffering of minor β-thalassemia have been found to possess higher, but not-significant, levels of IL-6 compared with healthy pregnancies (38). Regarding HT pregnant and non-pregnant women, they have been described with higher levels of IL-6 compared with healthy pregnancies (29, 38).

Here, we present the data on the efficacy of bLf oral administration in treating anemia in minor β-thalassemic pregnant and non-pregnant women as well as in the management of AI in pregnant and non-pregnant women affected by HT. The bLf efficacy is also reported in pregnant women suffering from other pathologies.

Remarkably, for the first time, we demonstrate that the efficacy of bLf in treating anemia and AI in β-thalassemic and HT women, respectively, can be correlated, in addition to the decrease to the IL-6, with two potential different molecular mechanisms: hepcidin-independent for β-thalassemic and hepcidin-dependent for HT women.

## Materials and methods

### Study design

To compare the efficacy of bLf treatment vs. the Italian ferrous sulfate one, usually applied worldwide as standard treatment, in the management of anemia and AI in pregnant and non-pregnant women affected by minor β-thalassemia and HT, respectively, a monocentric interventional clinical study has been conducted. In addition, the efficacy of bLf oral administration was also carried out on anemic pregnant women suffering from different pathologies as epilepsy, insulin resistance diabetes type 2, Crohn's disease, hypertension and HT (twin pregnancies).

### Setting

The interventional clinical study (ClinicalTrials.gov Identifier: NCT01221844) has been conducted at Clinica Fabia Mater, via Olevano Romano 25, Rome, Italy, a secondary-level hospital for complicated pregnancies, in accordance with the ethical principles of the Declaration of Helsinki and the Good Clinical Practice. Approval was granted by the Ethics Committee of Clinica Fabia Mater, via Olevano Romano 25, Rome, Italy (FM MOD 26022010). All women gave written informed consent before undergoing any study procedure. All women have been recruited from June 2010 to October 2013. All pregnant women were examined at the time of enrolment and every 30 days at the scheduled visits until delivery. All women of child-bearing age were examined at the time of enrolment and 30 days after treatments.

### Patients

A total of 90 anemic pregnant women between the 6th and 8th week of gestation has been enrolled, thereof 20 women with minor β-thalassemia and 70 with HT, and 20 anemic affected by other pathologies, including epilepsy (No 9), insulin resistance diabetes type 2 (No 3), Crohn's disease (No 3), hypertension (No 2), and HT twin pregnancies (No 3), enrolled at different weeks of gestation.

Pregnant women with uncomplicated pregnancy were excluded as well as if smokers or if they had anti-phospholipid syndrome, other concomitant diseases, infections, previous iron supplementation therapy, recent blood transfusion(s), obesity, and allergy to milk proteins, factors that could be considered potential modifiers of the treatments. In addition, this interventional clinical study included 88 anemic women of child-bearing age, thereof 9 affected by minor β-thalassemia and 79 by HT.

Non-pregnant women were excluded if smokers or if they had other concomitant diseases, infections, previous iron supplementation therapy, recent blood transfusion(s), obesity and allergy to milk proteins.

All enrolled HT pregnant and non-pregnant women received low molecular-weight heparin (0.3 U/day of Seleparina, Italfarmaco SpA, Milano-Italy) and low dose aspirin (100 mg every 2 days of Cardioaspirin® 100, Bayer SpA, Milano-Italy) to prevent and reduce the risk of venous thromboembolism and miscarriage associated to hypercoagulability (39).

### Blood analyses

Each blood sample was harvested, aliquoted, and stored at −80°C in order to analyze the parameters at different times. The anemia is defined when one of the hematological parameters corresponded to the following values: RBCs <4,000,000/ml, Hb <11 g/dl, TSI <30 μg/dl, and sFtn <12 ng/ml.

The diagnosis of β-thalassemia was carried out, at the laboratory of Clinica Fabia Mater, by the analysis of the hematological parameters including red cell morphology and indices that revealed microcytosis (low mean corpuscular volume, MCV), reduced content of Hb per red cell (low mean corpuscular hemoglobin, MCH), followed by separation and measurement of Hb fractions through electrophoresis (40).

Moreover, TSI and sFtn were assessed from venous blood. In particular, TSI was measured using an Iron FZ assay (Hoffmann-LaRoche, Basel, Switzerland) based on a guanidine hydrochloride/Ferrozine reaction, while sFtn was measured using a radioimmunoassay (Spectria, Orion Diagnostics). Serum IL-6 and hepcidin were also detected. Serum IL-6 levels were determined by standard ELISA Quantitative kits (R&D Systems, Wiesbaden, Germany) at Department of Public Health and Infectious Diseases, Sapienza University of Rome, Italy, on the samples collected before and after treatment. Serum hepcidin was detected according to competitive enzyme-linked immunosorbent assay (Intrinsic Life Sciences, La Jolla, CA) at Department of Public Health and Infectious Diseases, Sapienza University of Rome, Italy.

The diagnosis of HT was performed at Institute Regina Elena, Department of Medical Pathology, Rome, Italy according to Jackson et al. (41). In particular, pregnant women with a history of adverse outcomes including recurrent miscarriages, preterm birth, intrauterine growth restriction, were screened for the following HT markers before the enrolment: protein C, protein S, activated protein C resistance, antithrombin deficiencies, elevated coagulation factors, hyperhomocysteinemia, F5 R506Q (factor V Leiden) and F2 G20210A (prothrombin G20210A) mutations. When at least one of these HT markers was found, pregnant women were considered affected by HT. All HT women were further screened for hematological parameters as RBCs, Hb, TSI, and sFtn as well as for serum IL-6 and serum hepcidin before starting and after 30 days of the treatments.

Moreover, for all enrolled women, the values of hematocrit, glycemia, uricemia, bilirubin, glutamic oxaloacetic transaminase, glutamic pyruvic transaminase, cholesterol, triglyceride acid, and electrolytes were evaluated at each visit (data not shown).

### Clinical examination

To evaluate the putative adverse effects of the treatments in pregnant women, the main outcomes were based on the estimation of maternal gastrointestinal discomfort, nausea, vomiting, diarrhea, and constipation.

At each visit, fetal vital sign assessments were monitored by ultrasonographic measurements of intrauterine growth and through the amount of amniotic fluid, expressed as amniotic fluid index (AFI), an index for the fetal well-being (42). An AFI of ≤5 cm was considered oligohydramnios, 5–8 cm borderline, and from 8 to 24 cm normal (42).

At the delivery, new-born weight and the APGAR score were also registered. The APGAR score is a practical method to evaluate the physical condition of a new-born shortly after delivery. The Apgar score comprises five components: (1) color, (2) heart rate, (3) reflexes, (4) muscle tone, and (5) respiration, each of which is given a score of 0, 1, or 2. An APGAR score of 0–3 at 5–10 min of age is predictive of high morbidity and mortality, a score of 4–6 as moderately abnormal, while a value of 9–10 indicates that the infant is in the best possible condition.

The adverse effects in women of child-bearing age were evaluated by gastrointestinal discomfort, nausea, vomiting, diarrhea and constipation.

### Interventions

Pregnant and child-bearing age women affected by minor β-thalassemia and HT suffering from anemia and AI, respectively, were assigned to different Arms on the basis of a personal preference. As matter of fact, before starting the study patients choose by themselves which treatment to adhere to.

Women included in Arm A, C, E, and G received the treatment based on the oral administration of one capsule containing 100 mg of bLf plus excipients (Lattoglobina®, Italy) two times a day before meals, to avoid the protein degradation due to the low pH of gastric juice during digestion (about 1.5). Conversely, at pH about 4, characteristic of gastric juice before meals, 90% of the administered bLf arrives undigested to the intestine (43). The total amount of elemental iron supplied by Lattoglobina® capsules corresponded to about 70–84 μg/day, depending on the degree of iron saturation (20–30%). Women included in Arm B, D, F, and H received the standard treatment based on the oral administration of a tablet containing 329.7 mg of ferrous sulfate (Ferro-Grad®, Abbot Laboratories, USA) once a day during meal, in order to avoid a possible gastric intolerance of the drug, according to the Italian Standard of Care. The total amount of elemental iron supplied by Ferro-Grad® tablets corresponded to about 105 mg/day.

All pregnant women affected by different pathologies, previously described, received oral administration of one capsule containing 100 mg of bLf plus excipients (Lattoglobina®, Italy) twice a day before meals, while HT women with twin pregnancies three times a day, more efficient in increasing hematological parameters than the administration of bLf twice a day.

### Further laboratory analysis

The purity of the encapsulated bLf (Molecular Weight of about 80 kDa) was checked by SDS-PAGE and silver nitrate staining, while its concentration was assessed by UV spectroscopy on the basis of an extinction coefficient of 15.1 (280 nm, 1% solution). The bLf iron saturation was about 20–30% as detected by optical spectroscopy at 468 nm on the basis of an extinction coefficient of 0.54 (100% iron saturation, 1% solution). The total amount of elemental iron supplied by Lattoglobina® capsules corresponded to about 70–84 μg/day, depending on the degree of iron saturation (20–30%). LPS contamination of bLf, estimated by Limulus Amebocyte assay (Pyrochrome kit, PBI International), was equal to 0.7 ± 0.06 ng/mg of bLf (9, 43).

### Statistical analysis

Statistical analysis was carried out using the ANOVA test. *P*-values has been obtained comparing before and after 30 days of each treatment the number of RBCs, the concentration of Hb, TSI, sFtn, IL-6 and hepcidin.

## Results

### Demographics

**Figure 1:**
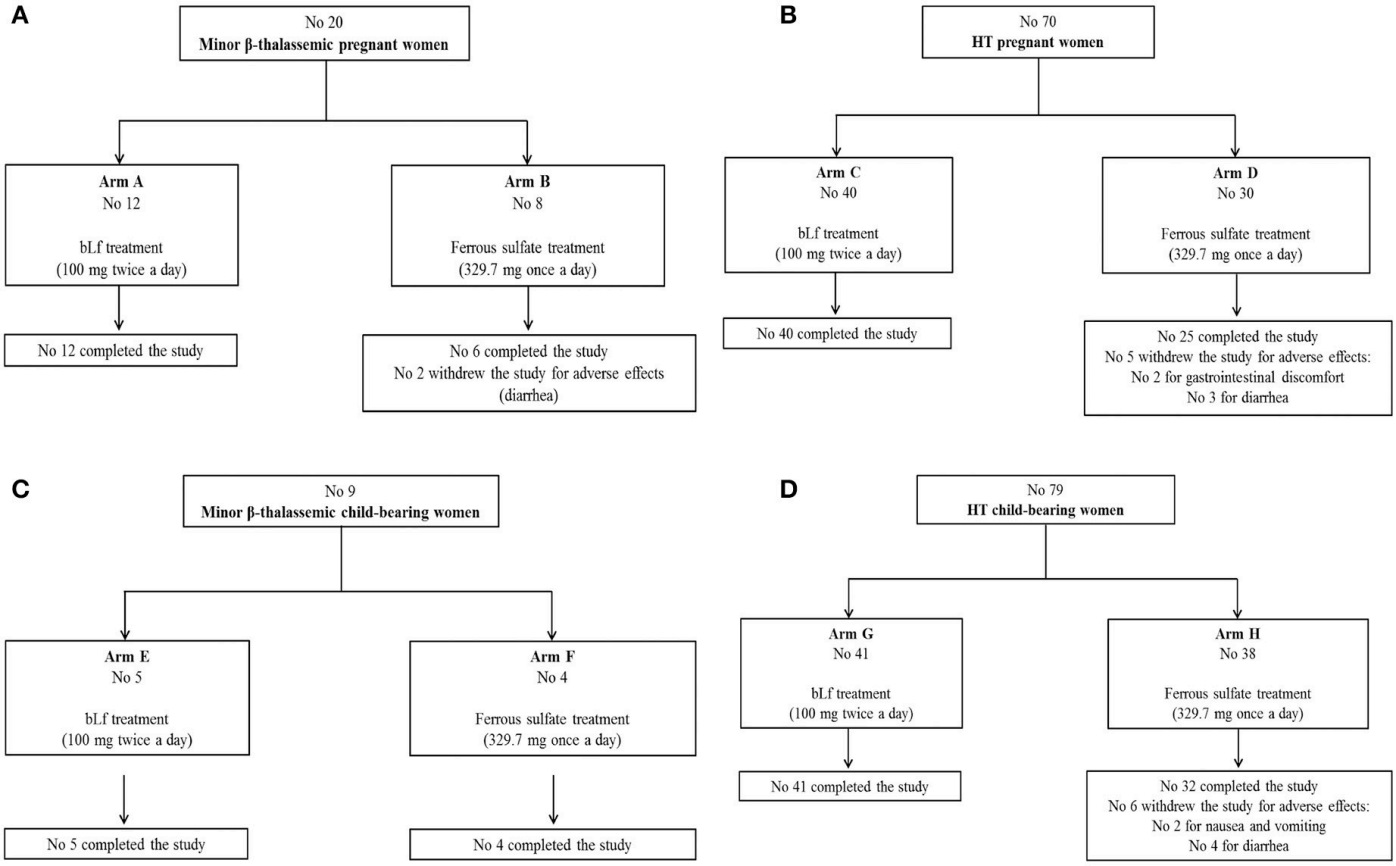
Flow diagrams of enrolled pregnant women suffering from minor β-thalassemia **(A)**, pregnant women suffering from hereditary thrombophilia **(B)**, women of child-bearing age suffering from minor β-thalassemia **(C)**, women of child-bearing age suffering from hereditary thrombophilia **(D)**.

A total of 20 minor β-thalassemic and 70 HT pregnant women suffering from anemia and AI, respectively, was enrolled in the interventional clinical trial within the first trimester of pregnancy (Figures 1A,B). On the basis of their personal preference, 12 and 8 minor β-thalassemic pregnant women were included in Arm A (bLf intervention) and Arm B (ferrous sulfate intervention), respectively (Figure 1A). Among 8 minor β-thalassemic pregnant women belonging to Arm B, 2 withdrew the study for adverse effects (diarrhea) (Figure 1A). Regarding HT pregnant women, on the basis of their personal preference, 40 were included in Arm C (bLf intervention) and 30 in Arm D (ferrous sulfate intervention) (Figure 1B). Among 30 HT pregnant women belonging to Arm D, 5 withdrew the study for adverse effects: 2 for gastrointestinal discomfort and 3 for diarrhea (Figure 1B). Demographics and hematological parameters of minor β-thalassemic and HT pregnant women, who completed the study, are reported in Table 1.

**Table 1:** Demographics and baseline of hematological mean values ± SD of minor β-thalassemic and HT pregnant women affected by anemia and AI, respectively, enrolled between the 6th and 8th week of gestation.

	**Minor β-thalassemic pregnant women**	**HT pregnant women**
No of pregnant women completing the study	18	65
Age (year)	28 ± 3	34 ± 3
Red blood cells x10^3^	*4, 470*±184	*3, 700*±145
Hemoglobin (g/dl)	7.7 ± 1.4	10.7 ± 0.6
Total serum iron (μg/dl)	50 ± 14	38 ± 13
Serum ferritin (ng/ml)	24 ± 4	14 ± 10
Serum IL-6 (pg/ml)	29 ± 9	87 ± 10
Hepcidin (ng/ml)	<1.0	111 ± 26

Moreover, 20 anemic pregnant women suffering from other pathologies, including epilepsy (No 9), insulin resistance diabetes type 2 (No 3), Crohn's disease (No 3), hypertension (No 2) and HT with twin pregnancies (No 3), have been enrolled at different trimesters of pregnancy and exclusively treated with bLf oral administration. Demographics as well as Hb and TSI values are reported in Table 2.

**Table 2:** Demographics and baseline of hemoglobin and total serum iron mean values ± SD of pathological pregnancies affected by anemia enrolled at different trimesters of gestation.

**No of women**	**Pathology**	**Age** **(year)**	**Week of** **pregnancy**	**Hb** **(g/dl)**	**TSI** **(μg/dl)**
9	Epilepsy	33 ± 4	17 ± 6	11.1 ± 0.6	42 ± 16
3	Insulin resistance diabetes type 2	35 ± 4	28 ± 4	10.4 ± 0.3	33 ± 2
3	Crohn's disease	31 ± 4	29 ± 4	9.4 ± 1.0	57 ± 7
2	Hypertension	34 ± 4	24 ± 2	10.7 ± 0.3	35 ± 5
3	HT (twin)	33 ± 2	25 ± 8	10.5 ± 0.5	47 ± 14

*Hb, hemoglobin; TSI, total serum iron*.

A total of 9 minor β-thalassemic and 79 HT women of child-bearing age suffering from anemia and AI, respectively, was also enrolled. On the basis of their personal preference, 9 minor β-thalassemic women of child-bearing age were divided in Arm E (No 5, bLf intervention) and Arm F (No 4, ferrous sulfate intervention), respectively, and completed the study (Figure 1C). Regarding HT women of child-bearing age, on the basis of their personal preference, 41 were included in Arm G (bLf intervention) and 38 in Arm H (ferrous sulfate intervention) (Figure 1D). Among 38 HT women belonging to Arm H, 2 withdrew the study for nausea and vomiting and 4 for diarrhea (Figure 1D).

Demographics and hematological parameters of enrolled women of child-bearing age are summarized in Table 3.

**Table 3:** Demographics and baseline hematological mean values ± SD of minor β-thalassemic and HT women of child-bearing age affected by anemia and AI, respectively.

	**Minor β-thalassemic women**	**HT women**
No of women completing the study	9	73
Age (year)	32 ± 2	31 ± 3
Red blood cells × 10^3^	*4, 287*±497	*3, 876*±247
Hemoglobin (g/dl)	10.6 ± 1.3	10.9 ± 0.8
Total serum iron (μg/dl)	41 ± 11	38 ± 11
Serum ferritin (ng/ml)	15 ± 5	10 ± 4
Serum IL-6 (pg/ml)	25 ± 13	43 ± 11
Hepcidin (ng/ml)	<1.0	102 ± 9

### Efficacy of bovine lactoferrin vs. ferrous sulfate treatment in pregnancy against anemia and AI

In pregnant women suffering from minor β-thalassemia and HT, the RBCs, Hb, TSI, sFtn, serum IL-6, and hepcidin were assessed before and every 30 days of treatments until delivery. The results obtained after the first 30 days of both interventions and at delivery, independently from the week of the gestation, are presented in the Tables 4, 5 for minor β-thalassemic and in Tables 6, 7 for HT pregnant women, respectively.

**Table 4:** Hematological parameters, serum IL-6 and hepcidin mean values ± SD of anemic minor β-thalassemic pregnant women treated with bovine lactoferrin (Arm A) or ferrous sulfate (Arm B), before and after 30 days of treatment.

	**Minor** β**-thalassemic pregnant women**			
	**Arm A**		**Arm B**	
**No of pregnant women completing the study Treatment**	**12**		**6**	
	**Bovine lactoferrin**		**Ferrous sulfate**	
	**Before**	**After 30 days**	**Before**	**After 30 days**
Red blood cells × 10^3^	*4, 487*±167	*4, 460*±121	*4, 453*±201	*4, 130*±123
Hemoglobin (g/dl)	7.8 ± 1.3	11.7 ± 0.8^*^	7.6 ± 1.6	10.2 ± 0.9^*^
Total serum iron (μg/dl)	54 ± 17	80 ± 9	46 ± 11	50 ± 12
Serum ferritin (ng/ml)	23 ± 5	28 ± 3	25 ± 3	24 ± 4
Serum IL-6 (pg/ml)	25 ± 8	6 ± 3^*^	33 ± 10	36 ± 8
Hepcidin (ng/ml)	<1.0	<1.0	<1.0	<1.0

* *Significantly different from before treatment (p < 0.001)*.

**Table 5:** Hematological parameters, serum IL-6 and hepcidin mean values ± SD of anemic minor β-thalassemic pregnant women treated with bovine lactoferrin (Arm A) or ferrous sulfate (Arm B), before the treatment and at delivery.

	**Minor** β**-thalassemic pregnant women**			
	**Arm A**		**Arm B**	
**No of pregnant women completing the study Treatment**	**12**		**6**	
	**Bovine lactoferrin**		**Ferrous sulfate**	
	**Before**	**At delivery**	**Before**	**At delivery**
Red blood cells × 10^3^	*4, 487*±167	*4, 610*±196	*4, 453*±201	*4, 284*±183
Hemoglobin (g/dl)	7.8 ± 1.3	11.9 ± 0.7^*^	7.6 ± 1.6	10.8 ± 0.6^*^
Total serum iron (μg/dl)	54 ± 17	87 ± 16	46 ± 11	55 ± 17
Serum ferritin (ng/ml)	23 ± 5	34 ± 6	25 ± 3	21 ± 7
Serum IL-6 (pg/ml)	25 ± 8	5 ± 4^*^	33 ± 10	34 ± 10
Hepcidin (ng/ml)	<1.0	<1.0	<1.0	<1.0

* *Significantly different from before treatment (p < 0.001)*.

**Table 6:** Hematological parameters, serum IL-6 and hepcidin mean values ± SD of HT pregnant women, suffering from AI, treated with bovine lactoferrin (Arm C) or with ferrous sulfate (Arm D), before and after 30 days of treatment.

	**HT pregnant women**			
	**Arm C**		**Arm D**	
**No of pregnant women completing the study Treatment**	**40**		**25**	
	**Bovine lactoferrin**		**Ferrous sulfate**	
	**Before**	**After 30 days**	**Before**	**After 30 days**
Red blood cells × 10^3^	*3, 750*±127	*4, 354*±198^*^	*3, 650*±163	*3, 960*±230
Hemoglobin (g/dl)	10.6 ± 0.4	12.2 ± 0.4^*^	10.8 ± 0.8	11.3 ± 1.1
Total serum iron (μg/dl)	36 ± 11	63 ± 9^*^	40 ± 15	40 ± 6
Serum ferritin (ng/ml)	11 ± 6	18 ± 2	17 ± 14	14 ± 9
Serum IL-6 (pg/ml)	89 ± 8	58 ± 6^*^	85 ± 12	108 ± 7
Hepcidin (ng/ml)	115 ± 23	65 ± 10^*^	107 ± 29	112 ± 32

* *Significantly different from before treatment (p < 0.001)*.

**Table 7:** Hematological parameters, serum IL-6 and hepcidin mean values ± SD of HT pregnant women, suffering from AI, treated with bovine lactoferrin (Arm C) or with ferrous sulfate (Arm D), before the treatment and at delivery.

	**HT pregnant women**			
	**Arm C**		**Arm D**	
**No of pregnant women completing the study Treatment**	**40**		**25**	
	**Bovine lactoferrin**		**Ferrous sulfate**	
	**Before**	**At delivery**	**Before**	**At delivery**
Red blood cells × 10^3^	*3, 750*±127	*4, 482*±147^*^	*3, 650*±163	*4, 029*±287
Hemoglobin (g/dl)	10.6 ± 0.4	12.7 ± 0.5^*^	10.8 ± 0.8	11.2 ± 0.9
Total serum iron (μg/dl)	36 ± 11	85 ± 10^*^	40 ± 15	38 ± 11
Serum ferritin (ng/ml)	11 ± 6	31 ± 3^*^	17 ± 14	12 ± 10
Serum IL-6 (pg/ml)	89 ± 8	50 ± 5^*^	85 ± 12	113 ± 15
Hepcidin (ng/ml)	115 ± 23	54 ± 13^*^	107 ± 29	116 ± 26

* *Significantly different from before treatment (p < 0.001)*.

As shown in Table 4, minor β-thalassemic pregnant women treated for 30 days with bLf (Arm A) showed a significant increase of Hb (from 7.8 ± 1.3 to 11.7 ± 0.8 g/dl) as well as a signficant decrease of IL-6 (from 25 ± 8 to 6 ± 3 pg/ml). Of note, a remarkable increase of TSI (from 54 ± 17 to 80 ± 9 μg/dl) was also observed. Conversely, minor β-thalassemic pregnant women treated for 30 days with ferrous sulfate (Arm B) showed a significant increase only for Hb (from 7.6 ± 1.6 to 10.2 ± 0.9 g/dl) while RBCs, TSI, sFtn and IL-6 levels remained unchanged (Table 4). Regarding serum hepcidin concentration, it was not detectable either before or after both treatments. At delivery, in the bLf-treated minor β-thalassemic women, in addition to a significant decrease of IL-6 (from 25 ± 8 to 5 ± 4 pg/ml), a significant increase of Hb (from 7.8 ± 1.3 to 11.9 ± 0.7 g/dl) as well as a remarkable increase of both TSI (from 54 ± 17 to 87 ± 16 μg/dl) and sFtn (from 23 ± 5 to 34 ± 6 ng/ml) were observed (Table 5). Conversely, in ferrous sulfate-treated minor β-thalassemic women, excepted for Hb concentration, no increase of other hematological parameters was detected (Table 5).

As shown in Table 6, HT pregnant women treated for 30 days with bLf (Arm C) showed a significant increase of RBCs (from 3,750 ± 127 × 10^3^ to 4,354 ± 198 × 10^3^), Hb (from 10.6 ± 0.4 to 12.2 ± 0.4 g/dl), TSI (from 36 ± 11 to 63 ± 9 μg/dl) and a slight increase of sFtn (from 11 ± 6 to 18 ± 2 ng/ml). These women presented high basal levels of IL-6 (89 ± 8 pg/ml) and hepcidin (115 ± 23 ng/ml) which were both significantly reduced (58 ± 6 pg/ml and 65 ± 10 ng/ml, respectively) already after 30 days of bLf treatment. Conversely, HT pregnant women treated for 30 days with ferrous sulfate (Arm D) showed a trend (*p* > 0.005), yet not significant, toward an increase of RBCs (from 3,650 ± 163 × 10^3^ to 3,960 ± 230 × 10^3^) and Hb (from 10.8 ± 0.8 to 11.3 ± 1.1 g/dl). Of note, both IL-6 and hepcidin levels do not show any change after the treatment.

At delivery, the bLf-treated HT women showed significant increases in all hematological values, such as RBCs (from 3,750 ± 127 × 10^3^ to 4,482 ± 147 × 10^3^), Hb (from 10.6 ± 0.4 to 12.7 ± 0.5 g/dl), TSI (from 36 ± 11 to 85 ± 10 μg/dl) and sFtn (from 11 ± 6 to 31 ± 3 ng/ml), as well as a significant decrease in both IL-6 and hepcidin levels (from 89 ± 8 to 50 ± 5 pg/ml and from 115 ± 23 to 65 ± 10 ng/ml, respectively). Conversely, HT women treated with ferrous sulfate management, showed no significant difference in any parameter evaluated compared to those obtained before the treatment (Table 7).

### Fetus and new-born outcomes

The adverse effects of bLf and ferrous sulfate treatments on fetuses were monitored through ultrasonographic measurements of intrauterine growth and by the detection of amniotic fluid amount, expressed as AFI (39). No growth restriction was observed for any fetus. The AFI ranged from 13.4 ± 1.6 mean values at 34 weeks to 10.5 ± 2.3 mean values at 40 weeks. It is interesting to note that all the values were within 8 to 24 cm range, which is accepted and established as normal range for AFI values (42).

The adverse effects on new-born were checked by new-born weight and APGAR score values. For both treatments, the new-born weight ranged among mean values corresponding to 2,950 ± 427 g for female and 3,478 ± 256 g for male. The APGAR score corresponded to range values of 8–10 for both sex, indicating new-born well-being.

### Efficacy of bovine lactoferrin treatment in pregnant women affected by various pathologies against anemia

Table 8 presents 20 anemic pregnant women affected by different pathologies including also 3 HT twin pregnancies. The treatment based on bLf oral administration appeared reasonably efficient in treating anemia by increasing both Hb and TSI concentrations. Even if the other hematological parameters were not evaluated, bLf is efficient in increasing these two parameters related to anemia. Interestingly, 9 patients suffering from epilepsy showed an increase of about 2- or 3-fold of TSI concentration and more than 1 g/dl of Hb. In 3 pregnant women affected by insulin resistance diabetes type 2, bLf intervention showed an increase of both Hb and TSI similarly to that observed in 3 pregnancies affected by Crohn's disease. The efficacy of bLf oral administration has been also observed in 2 women with hypertension during the gestation. Of note, a great efficacy has been also observed in 3 HT twin pregnancies treated with 100 mg bLf three times a day before meals (instead of two times a day usually applied).

**Table 8:** Mean values ± SD of hemoglobin and total serum iron of anemic pregnant women suffering from different pathologies treated for 30 days with bovine lactoferrin.

**No of women**	**Pathology**	**Age (year)**	**Week of pregnancy**	**Hb (g/dl)**		**TSI (**μ**g/dl)**	
				**Before**	**After 30 days**	**Before**	**After 30 days**
9	Epilepsy	33 ± 4	17 ± 6	11.1 ± 0.6	12.7 ± 0.4	42 ± 16	111 ± 24
3	Insulin resistance diabetes type 2	35 ± 4	28 ± 4	10.4 ± 0.3	11.4 ± 0.3	33 ± 2	56 ± 11
3	Crohn's disease	31 ± 4	29 ± 4	9.4 ± 1.0	10.6 ± 0.5	57 ± 7	67 ± 8
2	Hypertension	34 ± 4	24 ± 2	10.7 ± 0.3	11.4 ± 0.1	35 ± 5	70 ± 14
3	HT twin	33 ± 2	25 ± 8	10.5 ± 0.5	11.6 ± 0.3	47 ± 14	99 ± 13

*Hb, hemoglobin; TSI, total serum iron*.

### Efficacy of bovine lactoferrin vs. ferrous sulfate treatment in women of child-bearing age against anemia and AI

Minor β-thalassemic women of child-bearing age treated for 30 days with bLf oral administration (Arm E) showed a remarkable, yet not significant, increase of both RBCs (from 4,280 ± 484 × 10^3^ to 4,990 ± 323 × 10^3^) and TSI (from 43 ± 11 to 84 ± 11 μg/dl). Moreover, slight increases in Hb (10.2 ± 0.8 to 11.3 ± 0.9 g/dl) and sFtn (from 18 ± 3 to 26 ± 5 ng/ml) levels as well as a decrease of serum IL-6 (from 24 ± 12 to 13 ± 8 pg/ml) concentration have been observed (Table 9). Conversely, no parameter was modified after ferrous sulfate treatment (Arm F). In addition, as observed in minor β-thalassemic pregnancies, hepcidin concentrations in minor β-thalassemic women of child-bearing age was not detectable (<1 ng/ml). Both bLf and ferrous sulfate interventions did not exert any effect on hepcidin synthesis (Table 9).

**Table 9:** Hematological parameters, serum IL-6 and hepcidin mean values ± SD of anemic minor β-thalassemic women of child-bearing age treated with bovine lactoferrin (Arm E) or with ferrous sulfate (Arm F), before and after 30 days of treatment.

	**Minor** β**-thalassemic women**			
	**Arm E**		**Arm F**	
**No of women completing the study Treatment**	**5**		**4**	
	**Bovine lactoferrin**		**Ferrous sulfate**	
	**Before**	**After 30 days**	**Before**	**After 30 days**
Red blood cells × 10^3^	*4, 280*±484	*4, 990*±323	*4, 294*±510	*4, 460*±315
Hemoglobin (g/dl)	10.2 ± 0.8	11.3 ± 0.9	11.0 ± 1.8	12.2 ± 1.3
Total serum iron (μg/dl)	43 ± 11	84 ± 11	39 ± 11	40 ± 15
Serum ferritin (ng/ml)	18 ± 3	26 ± 5	12 ± 7	15 ± 5
Serum IL-6 (pg/ml)	24 ± 12	13 ± 8	26 ± 14	32 ± 11
Hepcidin (ng/ml)	<1.0	<1.0	<1.0	<1.0

Regarding HT women of child-bearing age, bLf treatment (Arm G) significantly increased the number of RBCs (from 3,664 ± 205 × 10^3^ to 4,280 ± 187 × 10^3^) and the concentration of Hb (10.4 ± 0.8 to 12.5 ± 0.6 g/dl), TSI (from 36 ± 10 to 90 ± 12 μg/dl) and sFtn (from 9 ± 3 to 22 ± 6 ng/ml). Furthermore, this treatment also significantly decreased serum IL-6 and hepcidin synthesis (Table 10), similarly to that observed in HT pregnancies.

**Table 10:** Hematological parameters, serum IL-6 and hepcidin mean values ± SD of HT women of child-bearing age, suffering from AI treated with bovine lactoferrin (Arm G) or with ferrous sulfate (Arm H), before and after 30 days of treatment.

	**HT women**			
	**Arm G**		**Arm H**	
**No of women completing the study Treatment**	**41**		**32**	
	**Bovine lactoferrin**		**Ferrous sulfate**	
	**Before**	**After 30 days**	**Before**	**After 30 days**
Red blood cells × 10^3^	*3, 664*±205	*4, 280*±187^*^	*4, 088*±289	*3, 989*±265
Hemoglobin (g/dl)	10.4 ± 0.8	12.5 ± 0.6^*^	11.4 ± 0.8	11.8 ± 1.0
Total serum iron (μg/dl)	36 ± 10	90 ± 12^*^	40 ± 12	45 ± 15
Serum ferritin (ng/ml)	9 ± 3	22 ± 6^*^	11 ± 5	14 ± 6
Serum IL-6 (pg/ml)	45 ± 9	9 ± 8^*^	41 ± 13	49 ± 8
Hepcidin (ng/ml)	100 ± 8	60 ± 18^*^	104 ± 10	100 ± 15

* *Significantly different from before treatment (p < 0.001)*.

Conversely, ferrous sulfate treatment in HT women of child-bearing age (Arm H) showed only a slight, yet not significant, increase for Hb and TSI. Of note, both IL-6 and hepcidin levels do not show any change after the treatment (Table 10).

## Discussion

Recently, Lf, a multifunctional glycoprotein, endowed with potent immune-modulatory properties, is emerging as an important component of iron and inflammatory homeostasis machinery (4, 23).

In 2006, by designing our first clinical trial on the effect of 30 days of bLf oral administration (100 mg two times a day before meals) compared with oral administration of ferrous sulfate (329.7 mg once a day during the meal) in anemic pregnant women, surprising results were obtained (36). Pregnant women receiving iron saturated bLf (20–30%) two times a day, absorbed 70–84 μg/day of elemental iron, respectively, through enterocytes. Although the concentration of iron supplemented by bLf is very far from that required daily (1–2 mg), a significant increase of the concentration of Hb and TSI was detected 30 days after treatment. Later on, in our subsequent clinical trials carried out on pregnant women suffering from anemia and AI, bLf treatment induced, in addition to Hb and TSI levels, a significant improvement of the number of RBCs and sFtn concentrations. Interestingly, a significant decrease of serum levels of IL-6, considered a key pro-inflammatory cytokine in iron homeostasis, was observed (4, 29, 37, 38). Differently from bLf, ferrous sulfate was ineffective in treating anemia and AI even if iron supply was a 1,000-fold higher than that furnished by bLf (about 100 mg/day vs. about 80 μg/day). Moreover, ferrous sulfate does not decrease the levels of serum IL-6 (4, 29, 36–38). Therefore, even if the mechanism by which bLf exerts its anti-inflammatory activity is still under debate, there are strong evidences that bLf efficacy in treating anemia and AI is not linked to a direct iron supplementation, but to a more complex mechanism involving this protein in decreasing IL-6 and modulating hepcidin and Fpn, the most important iron homeostasis actors, both regulated by IL-6 (23, 44).

In this respect, our group has recently demonstrated that bLf is able to revert the iron disorders in human inflamed-macrophages. In particular, bLf, by exerting its anti-inflammatory activity vs. IL-6 synthesis, determines the up-regulation of Fpn expression, thus leading to an efficient recovery of iron efflux from cells to blood (8, 9).

The bLf anti-inflammatory activity has been explained by the discovery of its nuclear localization thus suggesting that this molecule may be involved in the transcriptional regulation of some genes of host inflammatory response (4, 45, 46). Lf is, therefore, a key element not only in the host defense system (47–49) but also a pivotal glycoprotein able to treat anemia through the inhibition of the inflammatory response, especially in pregnant women affected by HT (29).

Here, we present novel and promising results on the efficacy and safety of bLf treatment in curing anemia and AI in minor β-thalassemic and HT pregnant and non-pregnant women, respectively, as well as anemia in pregnant women suffering from various pathologies.

For the first time, the serum hepcidin has been detected in minor β-thalassemic and HT pregnant and non-pregnant women and correlated with all the other clinical parameters evaluated before and after bLf or ferrous sulfate management.

Furthermore, we confirm the results obtained in our previous clinical trials on HT pregnancies (29) through the enrolment of additional 70 HT pregnant women and 79 HT women of child-bearing age.

Overall, both HT pregnant and non-pregnant women, after 30 days of bLf treatment, show a significant increase of RBCs, Hb, TSI and sFtn. Of note, bLf treatment also significantly reduces IL-6 and hepcidin levels compared to the high basal concentrations (Table 6 Arm C and Table 10 Arm G). The effect of bLf treatment, leading to the down-regulation of hepcidin synthesis, should be considered as a signal of a regulatory mechanism exerted by bLf to avoid the pathological intracellular iron overload, thus restoring the physiological iron export to blood through Fpn up-regulation. In fact, the persistence of high levels of IL-6 and hepcidin would inhibit the iron export through the down-regulation and degradation of Fpn, causing an unsafe intracellular iron overload, specially in those cells involved in iron absorption and recycle, such as enterocytes and macrophages, respectively. As matter of fact, inflammation-mediated iron retention in macrophages determines the most pivotal cause of AI establishment and maintenance. Indeed, upon inflammatory stimulation, macrophages polarize in a sub-population, namely M1, characterized by production of pro-inflammatory cytokines, such as IL-6, the down-regulation of Fpn and up-regulation of cytosolic Ftn, leading to the block of iron recycling to blood from senescent erythrocytes, the major iron source for the body (50, 51). The current finding that bLf, through its anti-inflammatory activity, is able to revert this unsafe condition restoring the physiological iron export from macrophages to blood can be considered an actual molecular mechanism to explain the bLf efficacy, iron independent, in treating AI in HT pregnant and non-pregnant women.

Conversely, ferrous sulfate treatment in HT pregnant and non-pregnant women shows a trend toward an increase in only RBCs and Hb. Moreover, this management fails in decreasing both IL-6 and hepcidin levels (Table 6 Arm D and Table 10 Arm H). The failure of ferrous sulfate treatment could be explained by its inability to modulate hepcidin or Fpn expression in a direct way (never demonstrated) or indirectly through the inhibition of IL-6 synthesis (never demonstrated).

The efficacy of bLf oral administration in treating anemia was also tested on pregnant women suffering from various pathologies. In these pregnant women bLf management appears effective (Table 8). Of note, regarding pregnant women affected by epilepsy, bLf treatment greatly increases TSI concentration (about 2- or 3-fold) and Hb (about 1 g/dl). The encouraging results were also obtained on HT twin pregnancies where the management has required an additional oral administration of bLf (100 mg three times a day).

In this interventional study, the efficacy of bLf oral administration has been also demonstrated in anemic pregnant and non-pregnant women affected by minor β-thalassemia, an inherited autosomal recessive Hb disorder characterized by reduced synthesis of the β-chain of Hb (24).

Even if the number of enrolled minor β-thalassemic women is low and does not allow to obtain conclusive and convincing results, bLf treatment demonstrates a simultaneous decrease in IL-6 and an increase in TSI both in pregnant and non-pregnant women even if at different extent (Table 4 Arm A and Table 9 Arm E). Remarkably, in these subjects hepcidin is not detectable both before and after bLf management. This observation supports the hypothesis that bLf, independently from hepcidin but dependently from IL-6 down-regulation, may act as a positive regulator of Fpn, thus increasing iron efflux to blood. This potential mechanism is indeed supported by the increase of TSI values in all minor β-thalassemic women already after 30 days of bLf treatment (Table 4 Arm A and Table 9 Arm E).

Similar to bLf, ferrous sulfate treatment in minor β-thalassemic pregnant and non-pregnant women leads to an increase of Hb concentration while, conversely to bLf, it does not influence IL-6 synthesis (Table 4 Arm B and Table 9 Arm F). Regarding serum hepcidin concentration, it was not detectable before and after ferrous sulfate intervention (Table 4 Arm B and Table 9 Arm F). Taken together all the results, alternatively to the hepcidin agonists or antagonists as therapeutic tools (20), bLf oral administration can be considered as a first promising compound in treating anemia through the decrease of both IL-6 and hepcidin thus restoring Fpn-mediated iron export from cells to blood in an hepcidin dependent or independent way.

Even if the number of patients is low and further investigations are required, these results highlight and support the importance of bLf oral administration in the treatment of the iron and inflammatory homeostasis disorders.

## Author contributions

PV and RP conceived, designed the clinical trial and wrote the first draft of manuscript. ML, LR, AC, and MC performed the laboratory analysis, analyzed the data, edited the manuscript, and prepared the figures and tables. All authors read and approved the final version.

### Conflict of interest statement

The authors declare that the research was conducted in the absence of any commercial or financial relationships that could be construed as a potential conflict of interest.
